# *Bifidobacterium animalis* subsp. *lactis* 832 Alleviates DSS-Induced Colitis in a Murine Model by Regulating Gut Microbiota and Phospholipid Metabolism

**DOI:** 10.3390/microorganisms14051090

**Published:** 2026-05-11

**Authors:** Xintong Chen, Qiushi Wang, Xiaoya Guo, Dan Li, Xinyu Wu, Xiaoya Li, Xiaoyu Zheng, Yangyang Li, Shuangshuang Han, Lu Feng, Bin Liu, Lei Wang

**Affiliations:** 1 National Key Laboratory of Intelligent Tracking and Forecasting for Infectious Diseases, TEDA Institute of Biological Sciences and Biotechnology, Nankai University, Tianjin 300457, China; cxtchenxinxin@outlook.com (X.C.); qiushiwenya@163.com (Q.W.); 18834820179@163.com (X.G.); lidancaas@163.com (D.L.); 1120230813@mail.nankai.edu.cn (X.W.); lixiaoyafighting@hotmail.com (X.L.); zhengxiaoyu_sdu@163.com (X.Z.); yyllyy0508@163.com (Y.L.); 2120241526@mail.nankai.edu.cn (S.H.); fenglu63@nankai.edu.cn (L.F.); 2 Key Laboratory of Molecular Microbiology and Technology, Nankai University, Tianjin 300457, China; 3 Nankai International Advanced Research Institute, Nankai University Shenzhen, Shenzhen 518045, China; 4 Southwest United Graduate School, Kunming 650092, China

**Keywords:** *Bifidobacterium animalis*, inflammatory bowel diseases, intestinal barrier integrity, gut microbiota, phosphatidylethanolamine, phosphatidylcholine

## Abstract

Inflammatory bowel disease (IBD) is a chronic intestinal disorder with recurrent inflammation for which effective therapeutic options remain limited. Probiotics from the *Bifidobacterium* genus have potential beneficial effects on the prevention of IBD by improving intestinal barrier integrity and modulating immune responses. However, whether these effects are mediated by the regulation of gut metabolism remains largely unclear. This study was designed to explore the protective effect of an infant-derived *Bifidobacterium animalis* subsp. *lactis* 832 (*B. lactis* 832) on dextran sulfate sodium (DSS)-induced colitis in mice and its underlying mechanism. *B. lactis* 832 treatment significantly alleviated colitis severity (*p* < 0.05), as evidenced by reduced weight loss, disease activity index (DAI), and colonic injury, accompanied by significantly decreased pro-inflammatory cytokine expression and increased *Il10* expression (*p* < 0.05). It also improved intestinal barrier integrity and modulated gut microbiota composition by reducing potentially pathogenic bacteria while enriching beneficial taxa. Surprisingly, metabolomic analysis revealed that *B. lactis* 832 intervention enhanced intestinal phospholipid metabolism, particularly increasing phosphatidylethanolamine (PE) and phosphatidylcholine (PC) levels. Notably, PE or PC supplementation recapitulated the protective effects against DSS-induced colitis (*p* < 0.05). These findings suggest that *B. lactis* 832 alleviates colitis through microbiota-associated metabolic regulation, highlighting a key role for phospholipid metabolism in mediating probiotic effects.

## 1. Introduction

Inflammatory bowel disease (IBD) is characterized by chronic and relapsing intestinal inflammation that includes ulcerative colitis (UC) and Crohn’s disease (CD) [1]. The clinical manifestations of IBD patients are diarrhea, blood in the stool, weight loss, and diffuse inflammation of the colonic mucosa [2]. IBD has become a prominent global healthcare problem, impacting more than 4 million individuals worldwide [3]. Although the pathogenesis of IBD is poorly understood, multiple lines of evidence suggest that the disease is caused by genetic susceptibility, intestinal barrier dysfunction, dysbiosis of the gut microbiota, and immune-mediated inflammation [4,5]. Recent studies have emphasized the crucial role of gut microbiota dysbiosis in the pathogenesis of IBD [6,7]. For instance, a reduced richness of microbial diversity, characterized by a decrease in Firmicutes and an overgrowth of Proteobacteria, is a typical hallmark of dysbiosis [8]. There is currently no cure for IBD and, despite available treatments, nearly half of patients develop refractory disease requiring ongoing management [9]. Given the complexity and appearing complications associated with immunosuppressive drugs (e.g., sulfasalazine and mesalazine) [10], it is crucial to develop novel therapeutic strategies and alternative treatments for IBD.

Considering the flexible manipulation of gut microbiota, potential remodeling by supplementing probiotics or providing specific substrates (such as food-derived nutrients) may provide a new method for the prevention of IBD [11]. Previous studies have shown that probiotic and prebiotic therapies are potentially natural and effective interventions for the treatment of IBD with fewer side effects than conventional therapies [12]. Among various probiotics, the *Bifidobacterium* genus exhibits multiple probiotic functions beneficial to gastrointestinal health, including repairing the intestinal barrier, modulating the intestinal microbiota, regulating the host immune system, and facilitating host nutrient absorption [13,14,15]. *Bifidobacterium animalis* subsp. *lactis* is one of the most extensively studied subspecies belonging to the genus *Bifidobacterium*. In vitro, strains of this subspecies present good probiotic properties, including high tolerance to acid and bile salts, strong adhesion ability, and potent anti-pathogenic activity [16,17,18]. In addition, some studies have confirmed that the *B. lactis* species had beneficial effects in alleviating colitis. For example, *B. lactis* XLTG11 has been reported to alleviate DSS-induced colitis by inhibiting the TLR4/MYD88/NF-κB signaling pathway [19]. Moreover, *B. lactis* A6 effectively alleviated DSS-induced colitis by preserving intestinal barrier integrity, reducing oxidative stress, and suppressing inflammatory responses [20]. However, the mechanisms underlying these protective effects are not fully understood, particularly whether they are mediated by microbiota-associated metabolic regulation.

Gut microbiota can influence host physiology through the production of bioactive metabolites, which play essential roles in immune regulation, signaling, and the maintenance of intestinal homeostasis [21,22]. Among these metabolites, phospholipids are critical components of intestinal mucosa with diverse biological functions, including supporting enterocyte proliferation, regulating lipid metabolism, and maintaining mucus secretion [23,24,25]. Phosphatidylcholine (PC) and phosphatidylethanolamine (PE) constitute the major phospholipid species in the intestinal mucosa, representing over 45% and around 25% of total phospholipids, respectively [26,27]. Dysregulation of phospholipid composition has been linked to the development of IBD. Reduced PC levels in patients with ulcerative colitis impair the hydrophobic and protective properties of the mucus layer [28,29]. In addition, PE has been reported to regulate mucus secretion by goblet cells and promote intestinal development [30]. These findings suggest that phospholipid metabolism may represent a key link between gut microbiota and intestinal barrier function in IBD.

In this study, we investigated whether an infant-derived *Bifidobacterium animalis* subsp. *lactis* strain (*B. lactis* 832) alleviated DSS-induced colitis in a mouse model by modulating microbiota-associated metabolic pathways. Special attention was directed toward uncovering critical metabolic pathways that connected microbial shifts with host protective effects. Furthermore, we aimed to characterize the interplay between microbial alterations and metabolic reprogramming to better understand the mechanistic basis of the protective effects of *B. lactis* 832.

## 2. Materials and Methods

### 2.1. Bacterial Strains

*Bifidobacterium animalis* subsp. *lactis* 832 (*B. lactis* 832), a strain isolated from infant feces in this study, was grown anaerobically at 37 °C in De Man, Rogosa and Sharpe medium (MRS) for 24–48 h. This strain was identified as *Bifidobacterium animalis* subsp. *lactis* based on 16S rRNA gene sequencing and phylogenetic analysis (GenBank accession no.: PZ135826; Figure S1 and Table S1). Fresh bacterial suspensions were prepared daily by centrifugation at 5500× *g* for 5 min at 4 °C, followed by two washes with phosphate-buffered saline (PBS, pH 7.4), and resuspension to approximately 5.0 × 10^9^ CFU/mL before administration.

### 2.2. Acid and Bile Tolerance Assay In Vitro

The survival of *B. lactis* 832 in a low pH environment and bile salt conditions was performed by evaluating viable colony counts as previously reported [18]. *B. lactis* 832 was grown overnight in MRS at 37 °C. For the acid tolerance assay, 1% (*v*/*v*) of the overnight bacterial culture was inoculated into the corresponding broth previously adjusted to pH 2.5 and 6.2 (control) with 1 N HCl. The samples from each tube of specified pH were taken at 0 and 4 h, and serially diluted. For the bile salt tolerance assay, 1% (*v*/*v*) of the overnight bacterial culture was inoculated into the corresponding broth supplemented with 0% (control) and 0.3% (*w*/*v*) oxgall bile salt (Sigma-Aldrich, St. Louis, MO, USA). The samples from each bile salt concentration group were taken at 0 and 24 h, and serially diluted. Cell counts for both assays were performed in triplicate by the standard plate count technique. The inoculated agar plates were incubated at the optimal temperature for 72 h, and the number of viable cells was calculated and expressed as the log_10_ value of CFU/mL. The survival rate of bacteria was calculated using the following equation [18].

Survival rate (%) = (log_10_ CFU/mL at a certain time point/log_10_ CFU/mL at 0 h) × 100

### 2.3. Caco-2 Cell Adhesion and Anti-Inflammatory Assay In Vitro

The methods for adherence and anti-inflammatory assays have been described previously [18,31]. For the adherence assay, Caco-2 cells were seeded into 6-well plates with Dulbecco’s Modified Eagle Medium (DMEM) containing 10% FBS at 37 °C with 5% CO_2_ and incubated until a complete monolayer was obtained. After washing with sterile PBS, bacteria were added at an MOI of 100 and co-incubated for 2 h. Nonadherent bacteria were removed by washing three times with PBS. The Caco-2 cells were then lysed with 0.5% Triton X-100 for 5 min. The lysates were diluted and plated on MRS agar and incubated anaerobically at 37 °C for 24–48 h. Adhesion efficiency was determined by counting the number of bacterial colonies. For the anti-inflammatory assay, after removing the culture medium, cells in 6-well plates were treated with *B. lactis* 832 at a concentration of 1 × 10^8^ CFU/mL for 2 h. Subsequently, all wells except the negative control were stimulated with LPS at a final concentration of 100 ng/mL. After an additional 24 h of incubation, total RNA was extracted, and the mRNA expression levels of TNF-α, IL-1β, IL-6, and IL-10 were determined by qRT-PCR.

### 2.4. Animal and DSS-Induced Colitis Model

Eight-week-old female C57BL/6J mice were obtained from Vital River Laboratory Animal Technology (Beijing, China). Animals were maintained under specific pathogen-free (SPF) conditions in the animal facility of Nankai University, with unrestricted access to standard chow and water, and kept on a 12 h light/dark cycle. All experimental procedures were approved by the Institutional Animal Care and Use Committee of Nankai University (Tianjin, China). All animal experiments were conducted in accordance with the guidelines of the Institutional Animal Care and Use Committee (IACUC) and were approved by the Animal Ethics Committee of Nankai University (Tianjin, China; Approval No. 2026-SYDWLL-000159).

**Figure 1:**
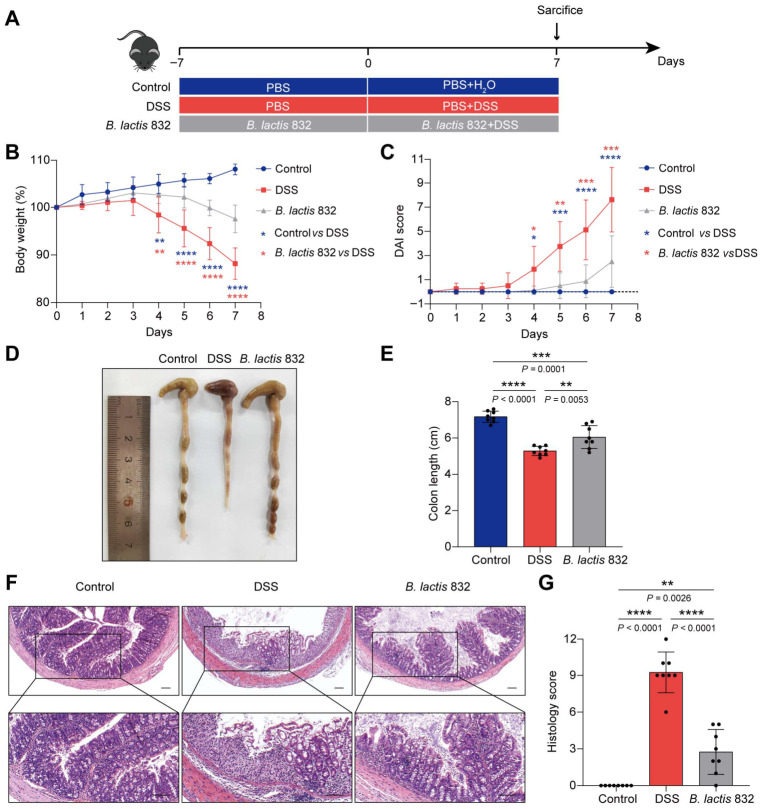
Effects of *B. lactis* 832 on DSS-induced colitis in mice. (**A**) Schematic of *B. lactis* 832 supplementation in DSS-induced mouse model. (**B**) Changes in body weight. (**C**) DAI score recorded from day 0 to day 7. (**D**) Gross morphology of the colon. (**E**) Quantification of colon length (cm). (**F**) Histological appearance of colon sections following H&E staining; scale bar: 100 μm. (**G**) Scoring of histological alterations in colonic tissues. *n* = 8. Statistical significance was determined using one-way ANOVA followed by Tukey’s multiple comparison tests (* p < 0.05; ** p < 0.01; *** p < 0.001; **** p < 0.0001).

The entire experiment lasted for 14 days, and the experimental design is presented in Figure 1A and Figure S3A, according to previous studies [14,32]. Following a 7-day acclimation period, mice were randomly assigned to different treatment groups. In the probiotic intervention experiment, animals were divided into four groups (*n* = 8 per group): Control, Control + *B. lactis* 832, DSS, and *B. lactis* 832. The control group received sterile water and PBS, whereas the DSS group was administered 3% (*w*/*v*) dextran sulfate sodium (DSS; 36–50 kDa, MeilunBio, Dalian, China) together with PBS. The Control + *B. lactis* 832 and *B. lactis* 832 group received sterile water or 3% DSS in combination with a daily oral gavage of 0.2 mL bacterial suspension (1 × 10^9^ CFU/mL). For PE or PC supplementation experiments, mice were divided into four groups (*n* = 8 per group): Control, DSS, PE, and PC. Animals in the control group were given sterile water and PBS, while DSS-treated mice received 3% DSS. The PE and PC groups were administered DSS along with daily gavage of PE or PC at a dose of 50 mg/kg (0.2 mL per mouse). Gavage treatment was maintained throughout the 14-day experimental period, whereas DSS exposure was limited to the last 7 days. Throughout the experiment, clinical parameters including body weight, stool consistency, and rectal bleeding were recorded to evaluate disease progression. The disease activity index (DAI) was calculated based on these parameters. Refer to Table 1 below for detailed scores [33]. The mice were euthanized on day 7, and the entire colon was excised, measured, and observed for signs of mucosal ulcers. A segment of the distal colon measuring 1 cm was collected for histological, AB-PAS, and immunofluorescence staining, the remaining colon and stools were snap-frozen in liquid nitrogen and stored at −80 °C for molecular analysis. Heart, liver, spleen, lung, and kidney tissues were harvested from mice in the Control and Control + *B. lactis* 832 groups for histopathological evaluation to assess biosafety [34].

### 2.5. Quantitative Reverse Transcription Polymerase Chain Reaction (qRT-PCR)

Total RNA was isolated from colon or ileal tissues using TRIzol reagent (GeneStar, Beijing, China) in accordance with the manufacturer’s protocol. All procedures were carried out under RNase-free conditions. First-strand cDNA was synthesized from RNA templates using oligo(dT) primers and StarScript Ⅲ MasterMix (GeneStar, Beijing, China). Quantitative real-time PCR (qRT-PCR) was performed on an Applied Biosystems 7500 system using 2× RealStar Fast SYBR qPCR mix (GeneStar). Relative gene expression levels were calculated using the 2^−ΔΔCt^ method with *Gapdh* serving as the internal reference gene [35]. The primer sequences used in this study are provided in Table 2.

### 2.6. Histological Assessment

Colon tissues were collected and rinsed with pre-chilled PBS to eliminate residual intestinal contents. Samples were subsequently fixed in 4% paraformaldehyde at room temperature for 72 h, followed by paraffin embedding. Paraffin blocks were sectioned using a Dakewe MT1 microtome (Dakewe Biotech, Shenzhen, China) and subjected to hematoxylin and eosin (H&E) as well as periodic acid–Schiff (AB-PAS) staining. Histological features were observed under a Leica DM2500 LED microscope (Leica, Wetzlar, Germany) at 100× magnification for pathological evaluation. Tissue damage was scored according to previously established criteria (Table 3) [36]. The number of goblet cells was quantified using ImageJ software (version 1.8.0.112; Media Cybernetics, Rockville, MD, USA).

### 2.7. Immunofluorescence Staining

Immunofluorescence staining was conducted following a standard procedure using primary antibodies against ZO-1 (1:500, #ab216880; Abcam, Cambridge, UK) and occludin (1:500, #FNab05957; FineTest, Wuhan, China). Tissue samples were incubated with the primary antibodies overnight at 4 °C, followed by washing with PBS. Subsequently, samples were incubated with DyLight 594-conjugated goat anti-rat IgG (H + L) secondary antibody (1:1000, E032440-01; EarthOx, San Francisco, CA, USA) for 1 h at 37 °C in the absence of light. After nuclear staining with DAPI, images were acquired using a laser scanning confocal microscope (ZEISS, Wetzlar, Germany). Quantification of ZO-1 and occludin fluorescence intensity was performed using ImageJ software (version 1.8.0.112, Media Cybernetics, Rockville, MD, USA).

### 2.8. Intestinal Permeability Assay

Intestinal barrier function was evaluated by assessing permeability to fluorescein isothiocyanate (FITC)-dextran following an established protocol. Briefly, mice were fasted for 4 h prior to the administration of 4 kDa FITC-dextran (Sigma-Aldrich, St. Louis, MO, USA) by oral gavage at 600 mg/kg body weight. After 3 h, blood samples were obtained via retro-orbital collection, and fluorescence intensity was determined at 525 nm using a microplate reader (BioTek, Winooski, VT, USA). Serum FITC-dextran levels were subsequently quantified based on a standard calibration curve.

### 2.9. 16S rRNA Sequencing Analysis

Fecal samples were collected from mice in different groups, immediately snap-frozen in liquid nitrogen, and submitted to Majorbio Bio-Pharm Technology Co., Ltd. (Shanghai, China) for 16S rRNA gene sequencing to analyze gut microbial composition. Microbial genomic DNA was isolated from feces using the HiPure Soil DNA Kit (cat. no. D3142-02B; Magen, Guangzhou, China). The V3–V4 hypervariable regions of the bacterial 16S rRNA gene were amplified using primer pairs 338F (5′-ACTCCTACGGGAGGCAGCAG-3′) and 806R (5′-GGACTACHVGGGTWTCTAAT-3′), followed by library preparation. Sequencing was carried out on Illumina MiSeq (PE300) or NovaSeq (PE250) platforms (Illumina, San Diego, CA, USA) according to standard procedures. Raw sequencing reads were first demultiplexed and subjected to quality control using fastp (v0.20.0), and paired-end sequences were subsequently merged with FLASH (v1.2.7). Operational taxonomic units (OTUs) were defined at a 97% sequence similarity threshold using UPARSE (v7.1), with chimeric sequences removed during processing. Taxonomic assignment of representative OTU sequences was performed using the RDP Classifier (v2.2) against the Silva 16S rRNA database (e.g., v138, https://www.arb-silva.de/) with a confidence cutoff of 0.7 [37,38]. All sequencing and bioinformatic analyses were performed on the Majorbio cloud platform (https://cloud.majorbio.com).

### 2.10. Metabolome Analysis

Fecal metabolomic profiling was performed using the Q300 Metabolite Assay Kit (Majorbio Bio-Pharm Technology Co., Ltd., Shanghai, China) with slight modifications to established protocols [1]. Briefly, approximately 50 mg of freeze-dried fecal material was weighed and homogenized in 400 μL methanol, followed by extraction with L-2-chlorophenylalanine (0.02 mg/mL) as an internal standard. The homogenates were centrifuged for 20 min, and 5 μL of the resulting supernatant was transferred into a 96-well plate for derivatization according to the manufacturer’s instructions. Metabolite quantification was carried out using an ultra-high-performance liquid chromatography system coupled to a Q Exactive HF-X mass spectrometer (Thermo Fisher Scientific, Waltham, MA, USA). Raw LC–MS data were processed using Progenesis QI software (version 3.0; Waters Corporation, Milford, CT, USA) for peak detection, alignment, and quantification. Differential metabolites were identified through univariate statistical analysis with a significance threshold of *p* < 0.05. For biomarker screening among the Control, DSS, and *B. lactis* 832 groups, metabolites meeting the criteria of |log_2_FC| ≥ 1 and *p* ≤ 0.05 in univariate analysis, along with a variable importance in projection (VIP) value > 1 from multivariate analysis, were considered significant. Data analysis was performed using the Majorbio cloud platform (https://cloud.majorbio.com).

### 2.11. Statistical Analysis

Data are expressed as the mean ± standard deviation (SD). Statistical analyses were performed by GraphPad Prism software (version 8.0.1; GraphPad Inc., San Diego, CA, USA). Differences between two groups were evaluated using an unpaired two-tailed Student’s *t* test, whereas comparisons among multiple groups were analyzed by one-way analysis of variance (ANOVA) followed by Tukey’s multiple comparison tests. A *p* value of less than 0.05 was considered indicative of statistical significance (* p < 0.05, ** p < 0.01, *** p < 0.001, **** p < 0.0001).

## 3. Results

### 3.1. In Vitro Characterization of B. lactis 832

Prior to in vivo experiments, we evaluated the probiotic properties of *B. lactis* 832. After treatment with simulated MRS (pH 2.5) for 4 h, the survival rate of *B. lactis* 832 was 78.99 ± 0.15% (Table S2). After exposure to 0.3% oxgall bile salt for 24 h, the survival rate remained 59.31 ± 0.12%, indicating good resistance to harsh gastrointestinal environments (Table S2). The adhesion rate of *B. lactis* 832 to Caco-2 cells was 40.92 ± 0.09%, demonstrating strong intestinal adhesion capacity (Table S2). Moreover, *B. lactis* 832 exerted prominent anti-inflammatory effects in vitro. Compared with the LPS-stimulated group, pre-treatment with *B. lactis* 832 significantly downregulated the mRNA expression of pro-inflammatory cytokines *Tnfa* (*p* < 0.0001), *Il1b* (*p* < 0.001) and *Il6* (*p* < 0.001), while markedly increasing the expression of the anti-inflammatory cytokine *Il10* (*p* < 0.01) (Figure S2). These in vitro probiotic properties confirm that *B. lactis* 832 is a reliable probiotic candidate for subsequent in vivo research.

### 3.2. B. lactis 832 Supplementation Alleviates DSS-Induced Colitis in Mice

Then, to investigate the prophylactic efficacy of *B. lactis* 832 against intestinal inflammation in vivo, we established a murine model of DSS-induced acute colitis (Figure 1A). Firstly, we administered 1 × 10^9^ CFU *B. lactis* 832 or PBS every day via oral gavage for two weeks and 3% DSS or H_2_O was added to the drinking water during the second week to induce colitis and the mice were sacrificed on day 7 after DSS treatment. Colitis severity was assessed daily using a composite DAI derived from body weight change, fecal bleeding, and stool consistency [2]. Continuous safety evaluation showed that single *B. lactis* 832 intervention caused no obvious physiological or histological abnormalities in healthy mice, with no significant differences in body weight, DAI score, colon length, or major organ histopathology between the normal control group and the *B. lactis* 832-alone treated group (*p* > 0.05) (Figure S3). The results showed that mice exposed to DSS exhibited greater weight loss and higher DAI scores than those in the control group (*p* < 0.05). While the *B. lactis* 832 group exhibited more modest weight loss and lower DAI scores than the DSS group (*p* < 0.05, Figure 1B,C). Moreover, colonic shortening may serve as an additional indicator for exacerbations of IBD [2]. On day 7, DSS-treated mice displayed a marked reduction in colon length relative to the control group (*p* < 0.0001), whereas *B. lactis* 832 administration partially restored colon length compared with the DSS group (*p* < 0.01, Figure 1D,E). Histological analysis showed an intact and well-organized mucosal structure without inflammatory cell infiltration in the control group. In contrast, DSS resulted in epithelial erosion, goblet cell loss, and areas of mucosal ulceration, together with increased leukocyte infiltration in the mucosa and submucosa, accompanied by higher histological scores of tissue damage and inflammation. However, the *B. lactis* 832 group exhibited less severe epithelial disruption, fewer damaged crypts, and reduced inflammatory cell infiltration resulting in a lower histological score compared to the DSS group (*p* < 0.001, Figure 1F,G). Taken together, these results demonstrated that *B. lactis* 832 supplementation reduced the severity of colitis symptoms in mice.

### 3.3. B. lactis 832 Supplementation Prevents DSS-Induced Colonic Inflammation

**Figure 2:**
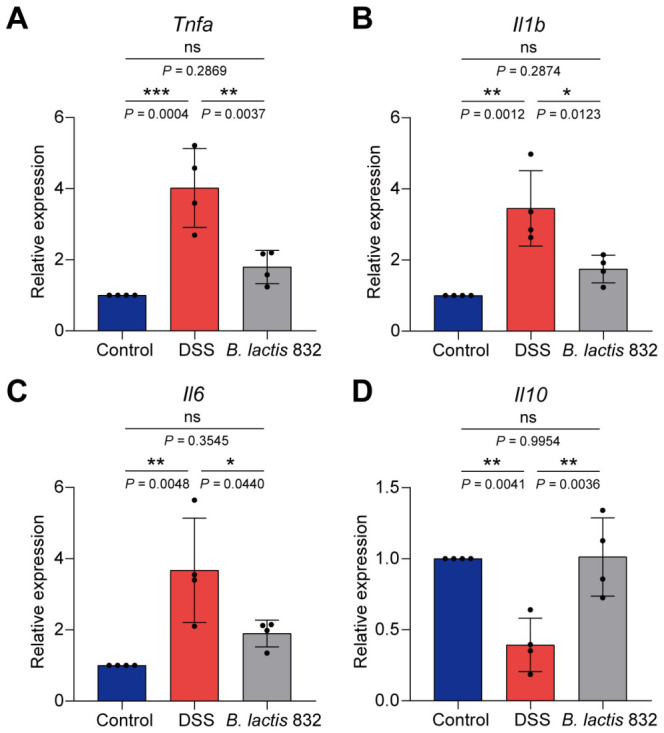
Effect of the *B. lactis* 832 on mRNA expression levels of *Tnfa*, *Il1b*, *Il6*, and *Il10* in DSS-induced colitis mice. (**A**–**D**) mRNA expression levels of *Tnfa*, *Il1b*, *Il6*, and *Il10*. *n* = 4. Statistical differences were evaluated by one-way ANOVA followed by Tukey’s multiple comparison tests (* p < 0.05; ** p < 0.01; *** p < 0.001).

To assess the impact of *B. lactis* 832 on intestinal inflammation, mRNA levels of *Tnfa*, *Il1b*, *Il6*, and *Il10* were quantified in colonic tissues following DSS exposure. DSS treatment was associated with elevated expression of pro-inflammatory cytokines (*Tnfa*, *Il1b*, and *Il6*) and reduced *Il10* levels compared with the control group (*p* < 0.01, Figure 2A–D). In contrast, administration of *B. lactis* 832 suppressed the upregulation of these pro-inflammatory markers while restoring *Il10* expression relative to the DSS group (*p* < 0.05). Collectively, these findings suggest that *B. lactis* 832 modulates cytokine profiles toward an anti-inflammatory state.

### 3.4. B. lactis 832 Attenuates DSS-Induced Mucus Impairment and Goblet Cell Depletion

**Figure 3:**
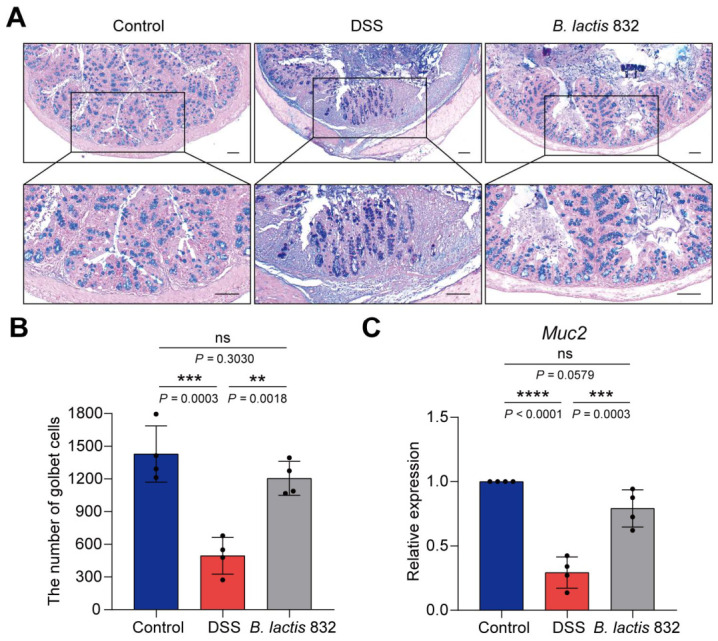
*B. lactis* 832 ameliorated DSS-induced mucus disruption and goblet cell exhaustion in DSS-induced colitis mice. (**A**) Representative Alcian blue and PAS staining of colon tissues. Scale bar: 100 μm. (**B**) Quantitative analysis of goblet cell abundance in the colon. (**C**) *Muc2* mRNA levels in colonic tissue. *n* = 4. Statistical significance was determined using one-way ANOVA followed by Tukey’s multiple comparison tests (** *p* < 0.01; *** *p* < 0.001; **** p < 0.0001).

Both clinical and experimental studies have implicated intestinal mucosal barrier dysfunction as a key factor underlying the pathogenesis and progression of IBD [8]. To further verify the alleviating effect of *B. lactis* 832 on the DSS-induced intestinal barrier disruption, the colon tissues were collected and AB-PAS staining and the RT-qPCR experiment were performed to analyze intestinal goblet cells contents and mucin secretion levels after DSS treatment. AB-PAS images showed the mucins were abundant in goblet cells and were mainly distributed on the surface of colonic epithelial cells. In contrast, the number of goblet cells was obviously decreased due to the damage to the inner and outer mucus layers of the colonic epithelium in the DSS group (*p* < 0.001). However, administration with *B. lactis* 832 effectively improved the loss of mucus layer and goblet cell counts, which showed protective effects on mucosa (*p* < 0.01, Figure 3A,B). Additionally, DSS treatment markedly reduced *Muc2* mRNA expression relative to control group (*p* < 0.0001), whereas *B. lactis* 832 supplementation restored its expression in colonic tissues (*p* < 0.001, Figure 3C). Above all, *B. lactis* 832 supplementation significantly improved mucus layer structure and increased goblet cell numbers in DSS-treated mice.

### 3.5. B. lactis 832 Supplementation Mediates Gut Barrier Function by Enhancing TJs Expression

**Figure 4:**
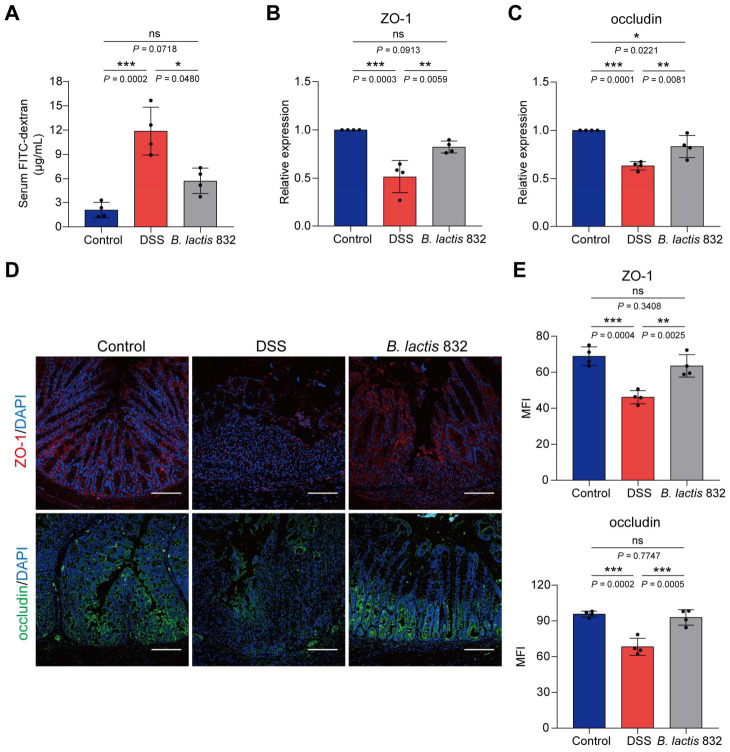
*B. lactis* 832 enhances intestinal barrier function in DSS-induced colitis. (**A**) Serum FITC-dextran levels as an indicator of intestinal permeability. (**B**,**C**) mRNA levels of ZO-1 (**B**) and occludin (**C**) in colonic tissue. (**D**,**E**) Immunofluorescence staining of ZO-1 (red) and occludin (green) with nuclear counterstaining (blue) in the colon (**D**), along with corresponding quantification (**E**). *n* = 4. Statistical significance was determined using one-way ANOVA followed by Tukey’s multiple comparison tests (* *p* < 0.05; ** *p* < 0.01; *** *p* < 0.001).

Gut barrier integrity was assessed by measuring intestinal permeability and the colonic expression of tight junction proteins, including ZO-1 and occludin, following DSS treatment. Firstly, intestinal permeability was assessed by measuring serum FITC-dextran levels 4 hours after administration for different groups on day 7 after DSS treatment. DSS treatment markedly increased serum FITC-dextran levels compared with control group (*p* < 0.001), while *B. lactis* 832 treatment attenuated this increase in serum FITC-dextran concentration (*p* < 0.05, Figure 4A). Consistently, mRNA levels of the tight junction proteins ZO-1 and occludin were reduced in DSS-treated mice (*p* < 0.001). In contrast, *B. lactis* 832 supplementation restored the expression of these genes relative to the DSS group (*p* < 0.01, Figure 4B,C). In addition, immunofluorescence staining confirmed that in situ protein expressions of ZO-1 and occludin in colon tissues were largely down-regulated after DSS treatment (*p* < 0.001), whereas *B. lactis* 832 treatment up-regulated their expression compared to the DSS group (*p* < 0.01, Figure 4D,E). Overall, *B. lactis 832* supplementation significantly restored the DSS-induced reduction in tight junction protein expression at both mRNA and protein levels.

### 3.6. B. lactis 832 Supplementation Improves the Intestinal Microbiota

**Figure 5:**
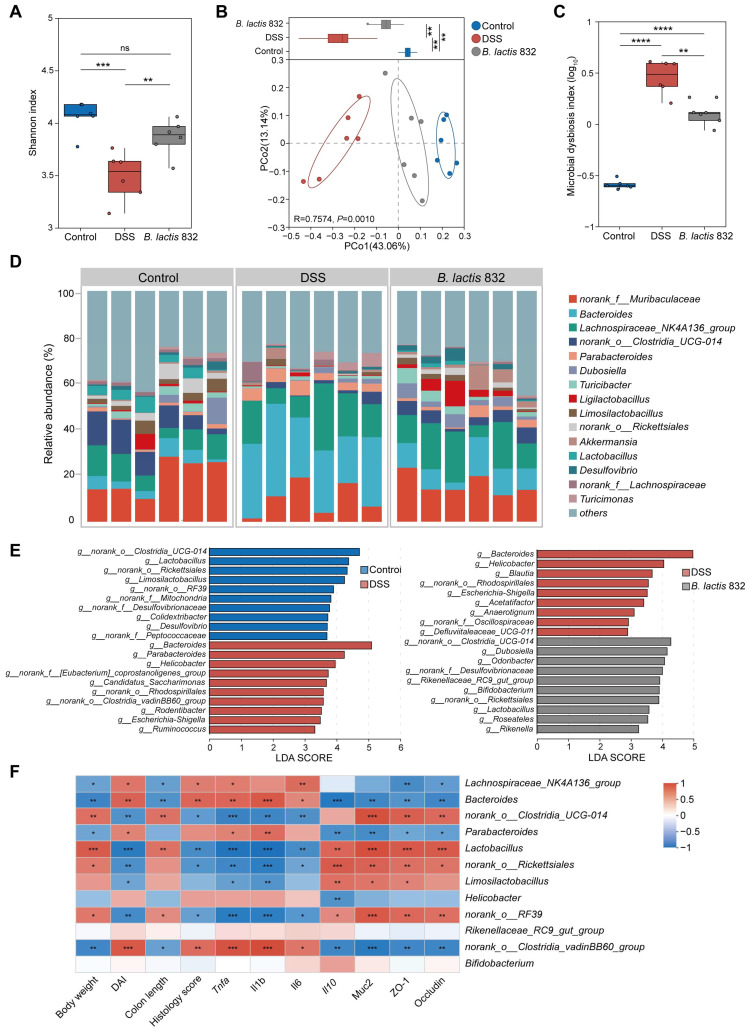
*B. lactis* 832 reshapes fecal microbial communities in DSS-induced colitis. (**A**) Shannon index reflecting alpha diversity of the gut microbiota. (**B**) Bray–Curtis-based principal coordinate analysis (PCoA) with PC1 scores (upper panels). (**C**) Microbial dysbiosis index (MDI) across Control, DSS, and *B. lactis* 832 groups. (**D**) Relative abundance of the top 15 genera in each group. (**E**) Differential taxa identified by LEfSe analysis between Control vs. DSS and *B. lactis* 832 vs. DSS groups (LDA score ≥ 2). (**F**) Spearman correlation heatmap showing associations between bacterial taxa and colitis-related biochemical parameters. *n* = 4–6. Statistical significance was determined using one-way ANOVA followed by Tukey’s multiple comparison tests (* p < 0.05; ** p < 0.01; *** p < 0.001; **** p < 0.0001).

Since gut dysbiosis is a pathological determinant of IBD, we investigated the regulatory effects of *B. lactis* 832 on the composition of gut microbiota using the 16S rRNA sequencing assay of fecal bacteria after DSS induced colitis. Amplicon sequencing of the 16S rRNA V3–V4 gene region yielded a total 1,328,283 high-quality reads (median 71,548.5 [IQR 5860.25] reads per sample) (Table S3). Rarefaction curves confirmed that sequencing depth was sufficient for reliable analysis (Figure S3). We firstly observed that the alpha diversity based on species richness and evenness information in OTU, shown by Shannon and Simpson index, showed a significant difference in the DSS group relative to the controls (*p* < 0.01), while *B. lactis* 832 intervention induced a larger Shannon index and a smaller Simpson index in colitis mice consistent with the control group (*p* < 0.01, Figure 5A and Figure S4). Principal coordinate analysis (PCoA) and non-metric multidimensional scaling (NMDS) based on Bray–Curtis distances revealed distinct clustering of microbial communities along the PC2 axis among the control, DSS, and *B. lactis* 832 groups, with the *B. lactis* 832 group positioned closer to the control group (*p* = 0.001, Figure 5B and Figure S5). The microbial dysbiosis index (MDI) was elevated in both DSS and *B. lactis* 832 groups relative to controls (*p* < 0.0001) but was lower in the *B. lactis* 832 group than in the DSS group (*p* < 0.01, Figure 5C). Subsequently, we conducted the linear discriminant analysis effect size (LEfSe) to discover the signature microbiota genera by assessing compositional differences between DSS and control groups, as well as between the DSS and *B. lactis* 832 groups. At the genus level, the control group significantly enriched *norank_o__Clostridia_UCG-014*, *Lactobacillus*, and *norank_o__Rickettsiales*, while *Bacteroides*, *Parabacteroides*, and *Helicobacter* were uniquely enriched in the DSS group (*p* < 0.05). However, *B. lactis* 832 supplementation dramatically enriched *norank_o__Clostridia_UCG-014*, *Dubosiella*, *Lactobacillus*, and *Bifidobacterium* compared to the DSS group (*p* < 0.05, Figure 5D,E and Table S4). Furthermore, we found that the signature bacterial genera of the DSS group had a positive correlation with the inflammatory indicators, in which *Bacteroides*, *Parabacteroides*, and *Helicobacter* had a positive correlation with *Tnfa*, *Il1b*, and *Il6* expression levels, DAI, and histology score, while these genera had a negative correlation with *Il10*, *Muc2*, *ZO-1*, and *occludin* expression levels, body weight and colon length (Figure 5F). Conversely, the signature bacterial genera of the control and *B. lactis* 832 groups, *norank_o__Clostridia_UCG-014*, *Lactobacillus*, and *norank_o__Rickettsiales*, had a positive correlation with *Il10*, *Muc2*, *ZO-1*, and *occludin* expression levels, body weight and colon length, while these genera had a negative correlation with *Tnfa*, *Il1b*, and *Il6* expression levels, DAI, and histology score (Figure 5F). Taken together, *B. lactis* 832 treatment improved the diversity and composition of gut microbiota in DSS-induced colitis mice.

### 3.7. Prophylactic B. lactis 832 Elevates Intestinal PE and PC Levels in DSS-Induced Colitis Mice

**Figure 6:**
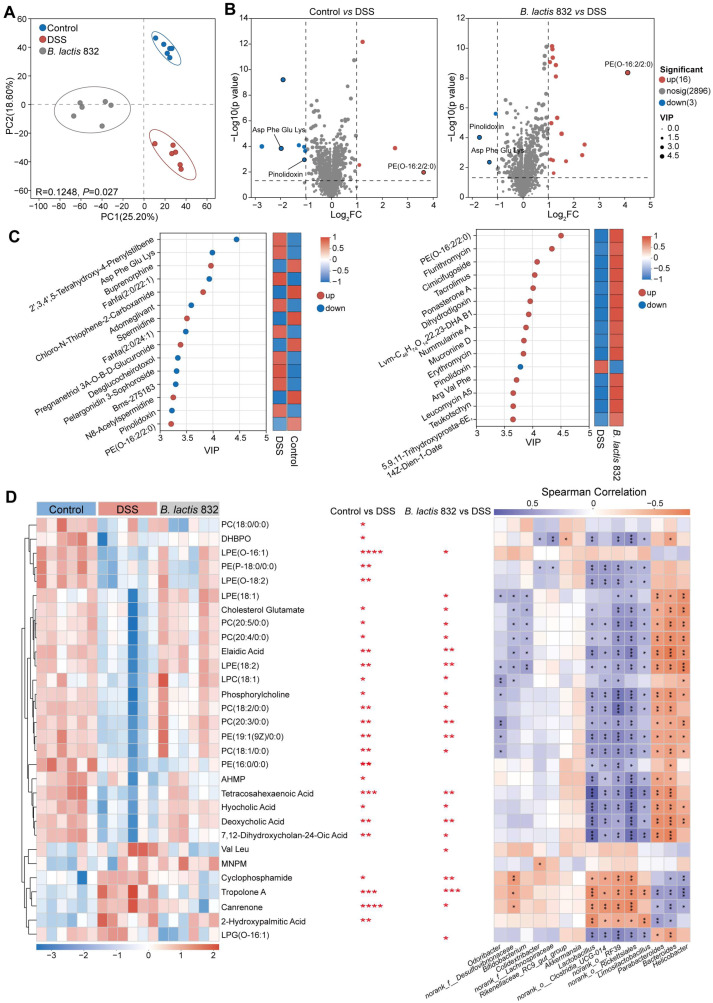
*B. lactis* 832 supplementation altered the composition of fecal metabolites of colitis mice. (**A**) Principal component analysis (PCA) showing group-wise differences in fecal metabolite profiles among Control, DSS, and *B. lactis* 832 groups. (**B**) Volcano plots displaying metabolites with significant changes (|log_2_FC| ≥ 1, *p* ≤ 0.05) in Control vs DSS and *B. lactis* 832 vs DSS groups. (**C**) Variable importance in projection (VIP) scores derived from the OPLS-DA model indicating the contribution of key metabolites to group separation. (**D**) Hierarchical clustering heatmap illustrating metabolite abundance patterns across groups (**left**), alongside Spearman correlation analysis between metabolites and gut microbiota (**right**). Color gradients represent relative metabolite levels, while correlation strength is reflected by color intensity (purple, positive; orange, negative). The asterisks (*, **, ***, ****) indicate the significance of metabolite abundance differences in the Control vs DSS and *B. lactis* 832 vs DSS groups. Long metabolite names are abbreviated in this figure for readability (DHBPO: (3R,6′Z)-3,4-Dihydro-8-hydroxy-3-(6-pentadecenyl)-1H-2-benzopyran-1-one; AHMP: (3β,5β,6α)-17-(acetyloxy)-3-hydroxy-6-methylpregnan-20-one; MNPM: (4-Methyl-3-Nitrophenyl)-(4-Methylpiperidin-1-Yl)Methanone). *n* = 6. Statistical significance was assessed using an unpaired two-tailed Student’s *t* test (* p < 0.05; ** p < 0.01; *** p < 0.001; ****p < 0.0001).

The biological effects of the gut microbiota after *B. lactis* 832 supplementation were further investigated through metabolomic analysis conducted on fecal samples after DSS induced colitis. The robust separation based on PCoA and PLS-DA analysis demonstrated the significant discrimination in metabolic profiles between the control, DSS and *B. lactis* 832 groups (*p* < 0.05, Figure 6A and Figure S6). Across the three groups, 1008 metabolites showed differential abundance. Using thresholds of |log_2_FC| ≥ 1 and *p* ≤ 0.05, 4 metabolites were upregulated and 7 were downregulated in the control group relative to the DSS group. In comparison, 16 metabolites were increased and 3 were decreased in the *B. lactis* 832 group versus the DSS group. Meanwhile, compared with the DSS group, PE (O-16:2/2:0) (phosphatidylethanolamine) was significantly up-regulated both in the control and *B. lactis* 832 groups, while Pinolidoxin and Asp Phe Glu Lys were significantly downregulated (Figure 6B). In addition, VIP calculated using OPLS-DA combined the differential accumulated metabolites analysis to identify the top 15 significant discriminant metabolites between the control and DSS groups, as well as between the DSS and *B. lactis* 832 groups. Compared with the DSS group, the VIP scores of up-regulated PE (O-16:2/2:0) were 3.2 and 4.5 in the control and *B. lactis* 832 groups, while the VIP scores of down-regulated Pinolidoxin were 3.2 and 3.8, respectively (Figure 6C). In addition, a heatmap was created to list and cluster the relative quantities of the top 30 fecal metabolites in the three groups. It was found that the relative abundance of phospholipid metabolic molecules, such as PC (18:0/0:0) (phosphatidylcholine), PE (P-18:0/0:0), and PE (16:0/0:0), were higher in the control group than in the DSS group (*p* < 0.05). Notably, *B. lactis* 832 supplementation, relative to DSS treatment, also significantly increased the relative abundance of molecules involved in phospholipid metabolism, such as (LPE (O-16:1) (lysophosphatidylethanolamine), LPC (18:1) (lysophosphatidylcholine), phosphorylcholine, PE (19:1(9Z)/0:0) and PC (18:1/0:0), as well as molecules related to secondary bile acid biosynthesis, including hyocholic acid and deoxycholic acid (*p* < 0.05, Figure 6D, Tables S5 and S6). To investigate the microbial factors responsible for these changes, a Spearman correlation analysis was conducted between the enriched metabolites and the bacterial taxa. Notably, *B. lactis* 832-enriched phospholipid metabolites, including PE and PC, were positively associated with beneficial bacteria such as *Bifidobacterium*, *Limosilactobacillus*, *norank_o__Rickettsiales*, *norank_o__RF39*, *norank_o__Clostridia_UCG-014*, and *Lactobacillus* (Figure 6D). Taken together, *B. lactis* 832 reshapes phospholipid metabolism in colitis mice, particularly increasing the intestinal PE and PC levels.

### 3.8. Exogenous PE and PC Supplementation Alleviates DSS-Induced Colitis in Mice

**Figure 7:**
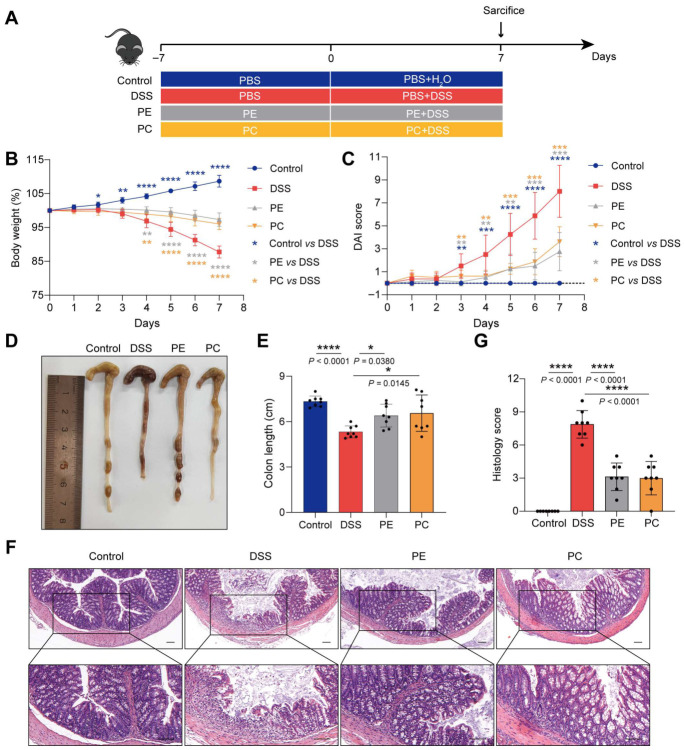
PE or PC supplementation alleviates DSS-induced colitis in mice. (**A**) Schematic of PE or PC supplementation in DSS-induced mouse model. PC: Phosphatidylcholine; PE: Phosphatidylethanolamine. (**B**) Changes in body weight. (**C**) DAI score recorded from day 0 to day 7. (**D**) Gross morphology of the colon. (**E**) Quantification of colon length (cm). (**F**) Histological appearance of colon sections following H&E staining Scale bar: 100 μm. (**G**) Scoring of histological alterations in colonic tissues. *n* = 8. Statistical significance was determined using one-way ANOVA followed by Tukey’s multiple comparison tests (* p < 0.05; ** p < 0.01; *** p < 0.001; **** p < 0.0001).

To further investigate whether the supplementation of *B. lactis* 832 improves colitis by promoting intestinal phospholipid metabolism, we directly supplemented PE or PC in the diet of mice to explore their effects on colitis (Figure 7A). Mice received daily oral gavage with PC or PE (50 mg/kg) or PBS for two weeks. Colitis was induced by administering 3% DSS in drinking water during the second week, while control mice received normal water. DSS treatment led to pronounced body weight loss and elevated DAI scores compared with the controls (*p* < 0.05). In contrast, mice supplemented with PC or PE exhibited attenuated weight loss and reduced DAI scores (*p* < 0.01, Figure 7B,C). Colon shortening was evident in DSS-treated mice (*p* < 0.0001), whereas PC or PE supplementation partially restored colon length (*p* < 0.05, Figure 7D,E). Histological analysis showed severe mucosal edema, epithelial disruption, crypt damage, and extensive inflammatory cell infiltration in the DSS group, resulting in higher histological scores (*p* < 0.0001). These pathological alterations were alleviated in mice receiving PC or PE, accompanied by lower histological scores (*p* < 0.0001, Figure 7F,G). Collectively, exogenous supplementation with PE or PC markedly relieved DSS-induced intestinal inflammation and pathological damage in colitis mice.

## 4. Discussion

Within microbiota-oriented interventions, probiotics are increasingly recognized as a promising option for the prevention and treatment of IBD [39]. *Bifidobacterium* have been well documented to alleviate colitis by reshaping gut microbiota, reinforcing intestinal barrier integrity and modulating host immune homeostasis [13,14,15,20,40]. Similarly, *B. lactis* 832 exerted consistent beneficial effects in this study, including mitigating intestinal inflammation and improving mucosal barrier function, and shared common microbial regulation characteristics with *Bifidobacterium longum* subsp. *infantis* FJSYZ1M3, *B. breve* and *B. lactis* BL-99, such as reducing the abundance of *Turicibacter* and *Bacteroides* in colitis mice [14,32,41]. Nevertheless, distinct microbial regulation patterns existed among different *B. lactis* strains. *B. lactis* 832 specifically enriched *norank_o__Clostridia_UCG-014* and *Dubosiella*, whereas *B. lactis* BL-99 increased the abundance of *Adlercreutzia*, and *B. lactis* XLTG11 markedly elevated *Muribaculaceae* levels in colitis mice [19,41]. Notably, most *Bifidobacterium* strains exert anti-colitis effects mainly by regulating short-chain fatty acid (SCFA) metabolism, whereas the core protective mechanism of *B. lactis* 832 is uniquely dependent on the remodeling of intestinal phospholipid metabolism, accompanied by significant increases in intestinal PE and PC contents [14,41,42]. These findings highlight the unique therapeutic potential of *B. lactis* 832 and its downstream phospholipid-dependent pathway, providing an innovative metabolic perspective for understanding probiotic function. Consistently, dietary supplementation with PE or PC recapitulated the protective effects, supporting a functional link between microbial modulation and host phospholipid metabolism [43,44]. Nevertheless, further clinical validation is still essential to verify its translational application in human IBD.

Mechanistically, the intestinal microbiome plays a fundamental role in the maintenance of gut homeostasis by regulating immune balance [45]. In our study, IBD-associated and pro-inflammatory taxa such as *Bacteroides*, *Parabacteroides*, and *Helicobacter* were enriched in the DSS group and positively correlated with the pathology of colitis and pro-inflammatory factors [46,47]. Notably, functional divergence exists among different species within the *Bacteroides* genus; not all members are pro-inflammatory, and certain strains such as *Bacteroides vulgatus* have been reported to alleviate colitis and maintain intestinal immune homeostasis [48]. Enterotoxigenic *Bacteroides fragilis* secretes enterotoxin (BFT, Bacteroides fragilis toxin) to elicit intestinal inflammation via E-cadherin cleavage/NF-κB signaling [49]. In addition, pathogenic *Helicobacter* spp., such as *Helicobacter muridarum* and *Helicobacter hepaticus*, have been proven to aggravate intestinal inflammation and elevate disease severity in multiple colitis models [6,50]. Moreover, IgA-coated *Helicobacter* and *B. fragilis* can penetrate into the mucous layer in gnotobiotic mice and exacerbate DSS-induced colitis [51]. Conversely, *B. lactis* 832 supplementation dramatically decreased the pro-inflammatory bacteria species, while effectively enriching beneficial commensals including *norank_o__Clostridia_UCG-014*, *Dubosiella*, *Lactobacillus*, and *Bifidobacterium*. *B. lactis* 832 may competitively exclude pathobionts by producing short-chain fatty acids (SCFAs) and lowering intestinal luminal pH, as well as support the metabolic cross-feeding of beneficial commensals, thereby stabilizing gut microbiota homeostasis under colitis conditions [42,52]. Notably, these enriched beneficial commensals negatively correlated with the pathology of colitis and pro-inflammatory factors in our study. Previous studies have shown that *Clostridium sporogenes*-derived metabolites, including IPA, SCFAs and BCFAs, increased the numbers of colonic tuft cells, the production of IL-22, and the polarization of Foxp3^+^ Tregs to alleviate colitis [53]. In addition, *Dubosiella newyorkensis* has a probiotic immunomodulatory effect on DSS-induced colitis, rebalancing Treg/Th17 responses and ameliorating mucosal barrier injury by producing SCFAs [54].

Emerging evidence suggests that gut microbiota can influence host physiology through the production and modulation of bioactive metabolites, which act as key mediators of microbe–host interactions [55,56]. Notably, phospholipid metabolism has been identified as a core pathway regulated by both gut commensals and pathogenic microbes, mainly through modulating phospholipase-mediated PC hydrolysis and *plsC*-dependent phospholipid synthesis [57,58,59]. On the one hand, *B. lactis* 832 may effectively inhibit excessive PC hydrolysis in the gut by reducing the relative abundance of *Helicobacter*. These pathobionts exhibit high ectophospholipase activity, including secreted phospholipase A2 (sPLA2), which efficiently degrades mucosal PC and disrupts the intestinal barrier [60,61]. On the other hand, *Bifidobacterium* and *Lactobacillus* species enriched by *B. lactis* 832 exhibit relatively low phospholipase activity, which may reduce the excessive hydrolysis of intestinal PC and PE [61,62,63]. Meanwhile, these beneficial commensals may possess a strong capacity to generate key precursors such as fatty acids, lysophospholipids, choline, and ethanolamine, thereby supporting host PC and PE synthesis and maintaining intestinal phospholipid homeostasis [64,65,66]. Correlation analysis further indicated that *Lactobacillus* was positively correlated with PC levels and could suppress plc-mediated PC hydrolysis, thereby promoting PC accumulation in the intestinal mucosa [59]. Notably, PC and PE are the predominant phospholipids in the intestine and are essential for mucosal integrity. Intestinal epithelial cells secrete mucus that forms a protective surface layer supported by a PC–PE bilayer structure [28,65]. Their depletion disrupts mucus integrity, exacerbates inflammation, and promotes disease progression in IBD [29]. Mechanistically, PE maintains lipid homeostasis and inhibits ferroptosis in a GPX4-independent manner, thereby protecting against colitis, while PC supports mucosal integrity when properly balanced [67]. Consistent with these roles, dietary PC alleviates DSS-induced colitis by improving barrier function, reducing inflammatory cytokines, and modulating gut microbiota composition [43,44]. This may also involve alterations in host metabolic pathways, including tryptophan and arginine metabolism [43]. Taken together, these findings suggest that PC and PE play essential and non-redundant roles in protecting against colitis. Restoration of phospholipid metabolism may represent a key mechanism underlying the anti-colitis effects of probiotics. The enrichment of beneficial bacteria, including members of *Bifidobacterium* and *Lactobacillus*, may contribute to this metabolic reprogramming and intestinal homeostasis. Collectively, these findings support a model in which *B. lactis* 832 alleviates colitis through a microbiota–phospholipid metabolism axis, thereby improving intestinal barrier function and modulating immune responses.

## 5. Conclusions

In conclusion, *B. lactis* 832 could effectively alleviate DSS-induced colitis symptoms in a murine model. The main mechanisms of *B. lactis* 832 that significantly alleviated DSS-induced colitis include inhibiting pro-inflammatory factors, maintaining intestinal barrier integrity, and improving the composition of gut microbiota and metabolomics. Multi-omics analysis further revealed that prophylactic *B. lactis* 832 enriched beneficial commensals *norank_o__Clostridia_UCG-014*, *Dubosiella*, *Lactobacillus*, and *Bifidobacterium* and enhanced intestinal levels of phospholipid metabolites, particularly phosphatidylethanolamine (PE) and phosphatidylcholine (PC). Furthermore, exogenous PE and PC administration recapitulated the protective effects of *B. lactis* 832 against colitis, supporting the critical role of phospholipid metabolism in mediating its probiotic function. A limitation of this study is the lack of direct microbial functional validation, which will be addressed in future metagenomic investigations to identify key strains and enzymes involved in phospholipid regulation. Nonetheless, our findings demonstrate that *B. lactis* 832 protects against colitis through a microbiota–phospholipid metabolism axis, supporting its potential as an innovative probiotic for IBD management. Future studies should include targeted gene knockout, mechanistic validation, and clinical trials to confirm its efficacy and safety. Collectively, this study provides preclinical evidence and a theoretical basis for developing microbiota-based strategies for IBD prevention and treatment.

## Figures and Tables

**Table 1:** Disease activity index.

Score	Weight Loss (%)	Stool Consistency	Bloody Stool Score
0	None	Normal	Normal colored stool
1	1–5	Loose stool	Brown stool
2	5–10	Loose stool	Reddish stool
3	10–15	Diarrhea	Bloody stool
4	>15	Diarrhea	Gross bleeding

**Table 2:** The primers used for qRT-PCR.

Genes	Forward (5′-3′)	Reverse (5′-3′)
**Human ^a^**		
*Gapdh*	TGCACCACCAACTGCTTAGC	GGCATGGACTGTGGTCATGAG
*T* *nfa*	ACAAGCCTGTAGCCCATGTT	AAAGTAGACCTGCCCAGACT
*I* *l1b*	GGATATGGAGCAACAAGTGG	ATGTACCAGTTGGGGAACTG
*I* *l* *6*	AGACAGCCACTCACCTCTTCAG	TTCTGCCAGTGCCTCTTTGCTG
*I* *l* *10*	CCTGGTGAAGACGTTTTGCA	TCATTCATGGCCTTGTAGACAC
**Mouse ^a^**		
*Gapdh*	ATGGCCTTCCGTGTTCCTAC	CAGATGCCTGCTTCACCAC
*T* *nfa*	AGACACCATGAGCACAGAAAGC	CCATAGAACTGATGAGAGGGAGG
*I* *l1b*	ATGGCAACTGTTCCTGAACTCAACT	CAGGACAGGTATAGATTCTTTCCTTT
*I* *l* *6*	CTAGGTTTGCCGAGTAGATCT	CACAAAGCCAGAGTCCTTCAGAGA
*I* *l* *10*	CCCTTTGCTATGGTGTCCTT	TGGTTTCTCTTCCCAAGACC
*Muc2*	CACCAACACGTCAAAAATCG	GGTCTCTCGATCACCACCAT
*ZO-1*	CAGAGTGGGGAAACCTCCATAG	GCGTTTTTCCCACTCTTCCT
*Occludin*	ATGTCCGGCCGATGCTCTC	TTTGGCTGCTCTTGGGTCTGTAT

^a^ Bold text is used only to clearly distinguish between human and mouse primer groups.

**Table 3:** Histological scores of colon damage.

Score	Inflammation Severity	Inflammation Extent	Crypt Damage	Percent Involvement
0	None	None	None	0%
1	Mild	Mucosa	Basal 1/3 damaged	1~33%
2	Moderate	Mucosa and submucosa	Basal 2/3 damaged	34~66%
3	Severe	Transmural	Crypt and surface epithelium lost	67~100%

## Data Availability

16S rRNA sequencing data and analysis codes are available in the NCBI-SRA database (BioProject: PRJNA1227203). Metabolomics data have been deposited in the NGDC-OMIX database (BioProject: PRJCA050718).

## Supplementary Materials

The following supporting information can be downloaded at: https://www.mdpi.com/article/10.3390/microorganisms14051090/s1, Figure S1: The phylogenetic tree was constructed by the DNA sequence of *Bifidobacterium animalis* 16S using MEGA 7 with the method of bootstrap consensus tree. The Sequence1, *B. lactis* 832, and other aligned species are marked with red and blue circles; Figure S2: Effect of the *B. lactis* 832 on mRNA expression levels of *Tnfa*, *Il1b*, *Il6*, and *Il10* in LPS-stimulated Caco-2 cells. *n* = 4. Statistical differences were evaluated by one-way ANOVA followed by Tukey’s multiple comparison tests (** p* < 0.05; *** p* < 0.01; **** p* < 0.001; ***** p* < 0.0001); Figure S3: Safety evaluation of *B. lactis* 832 supplementation in mice. (A) Schematic of *B. lactis* 832 supplementation in mouse model. (B) Changes in body weight. (C) DAI score recorded from day 0 to day 7. (D) Gross morphology of the colon. (E) Quantification of colon length (cm). (F) Histological appearance of colon sections following H&E staining. Scale bar: 100 μm. (G) Scoring of histological alterations in colonic tissues. (H) Representative images of major organs (heart, liver, spleen, lung, kidney) stained with H&E staining. Scale bar: 100 μm. *n* = 8. Statistical significance was determined using one-way ANOVA followed by Tukey’s multiple comparison tests (ns *p* > 0.05); Figure S4: Rarefaction curves of observed species for all samples; Figure S5: Alpha diversity of the gut microbiota analysis by the Simpson index (*n* = 6). Figure S6: Non-metric Multidimensional Scaling (NMDS) and scores of the PC1 axis (upper panels) of Bray–Curtis dissimilarity based on OTUs; Figure S7: Partial Least Squares Discriminant Analysis (PLS-DA) illustrating changes in the composition of the fecal metabolites among Control, DSS and *B. lactis* 832 groups; Table S1: Identification of *Bifidobacterium animalis* subsp. *lactis* strain 832 based on 16S rRNA gene sequence similarity. Table S2: Determination of key in vitro probiotic traits of *B. lactis* 832; Table S3: Statistics of Sample 16S rRNA Sequencing Information; Table S4: Gut microbiota composition among Control, DSS and *B. lactis* 832 groups at the genus level; Table S5: Differentially expressed metabolites between Control and DSS groups; Table S6: Differentially expressed metabolites between *B. lactis* 832 and DSS groups.
