# Supplementary material for: Bifidobacterium animalis subsp. lactis 832 Alleviates DSS-Induced Colitis in a Murine Model by Regulating Gut Microbiota and Phospholipid Metabolism
Source: Microorganisms. 2026 May 11;14(5):1090. doi: 10.3390/microorganisms14051090 (PMC13210333; doi:10.3390/microorganisms14051090)
Supplement: Supplementary file 1 [file microorganisms-14-01090-s001.zip › microorganisms-4256083-supplementary/Supplementary Figure.pdf]

S1

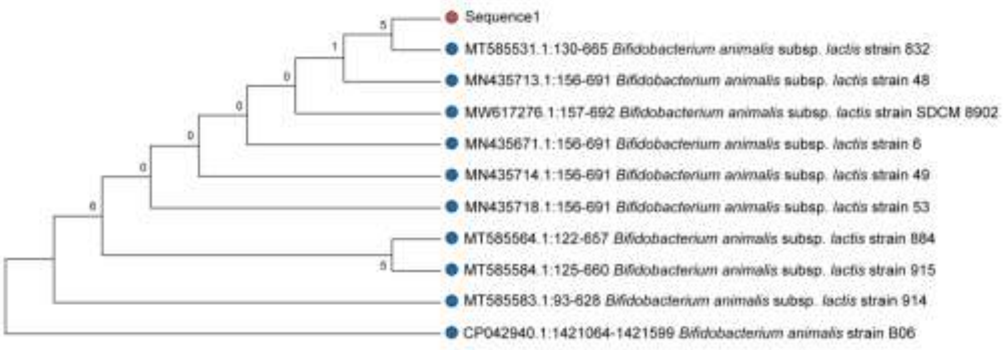

Figure S1. The phylogenetic tree was constructed by the DNA sequence of *Bifidobacterium animalis* 16S using MEGA 7 with the method of bootstrap consensus tree. The Sequence1, *B. lactis* 832, and other aligned species are marked with red and blue circles.

S2

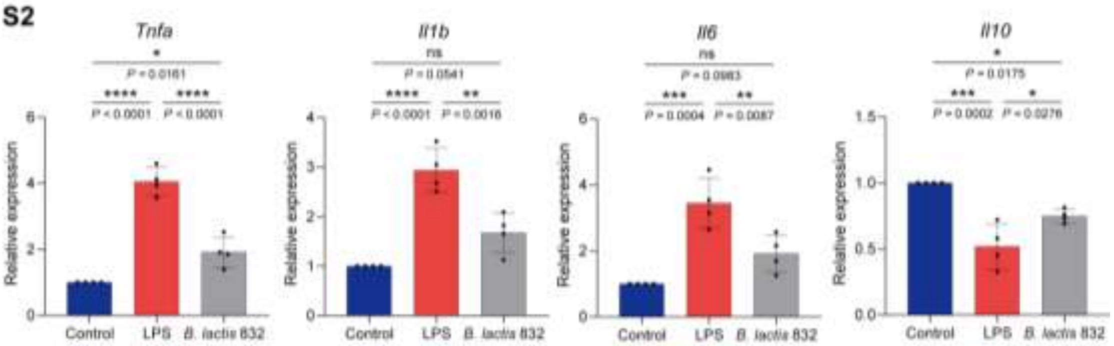

Figure S2. Effect of the *B. lactis* 832 on mRNA expression levels of *Tnfa*, *Il1b*, *Il6*, and *Il10* in LPS-stimulated Caco-2 cells. $n = 4$. Statistical differences were evaluated by one-way ANOVA followed by Tukey's multiple comparison tests (*$p < 0.05$; **$p < 0.01$; ***$p < 0.001$; ****$p < 0.0001$).

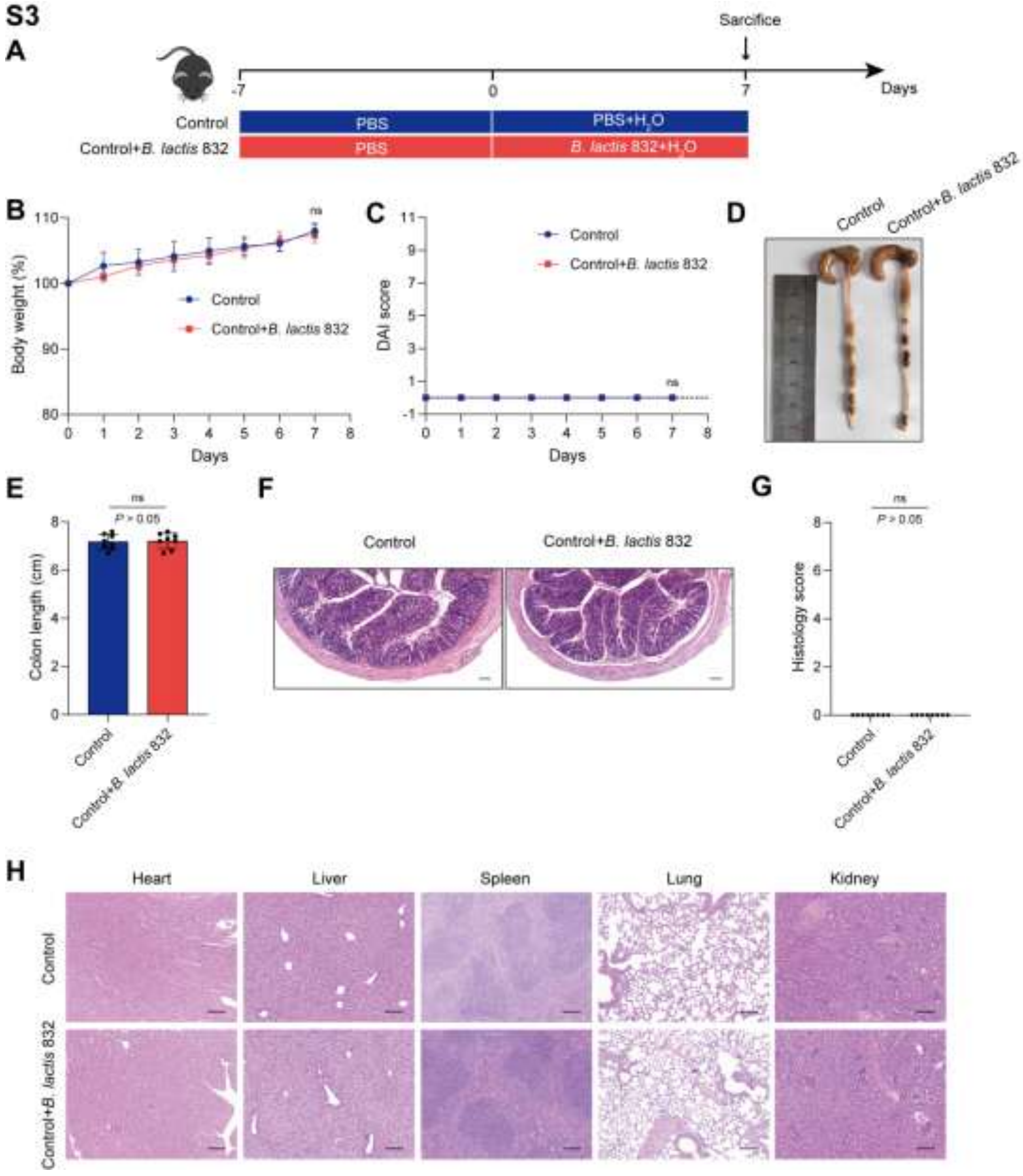

Figure S3. Safety evaluation of *B. lactis* 832 supplementation in mice. (A) Schematic of *B. lactis* 832 supplementation in mouse model. (B) Changes in body weight. (C) DAI score recorded from day 0 to day 7. (D) Gross morphology of the colon. (E) Quantification of colon length (cm). (F) Histological appearance of colon sections following H&E staining Scale bar: 100 µm. (G) Scoring of histological alterations in colonic tissues. (H) Representative images of major organs (heart, liver, spleen, lung, kidney) stained with H&E staining Scale bar: 100 µm. n = 8. Statistical significance was determined using one-way ANOVA followed by Tukey's multiple comparison tests (ns $p > 0.05$).

S4

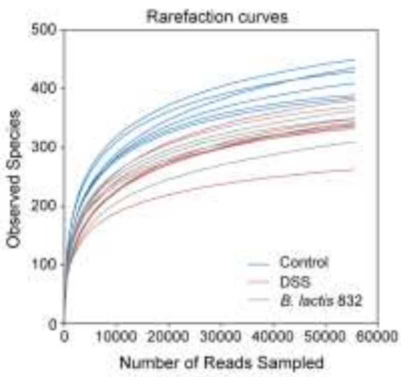

Figure S4. Rarefaction curves of observed species for all samples.

S5

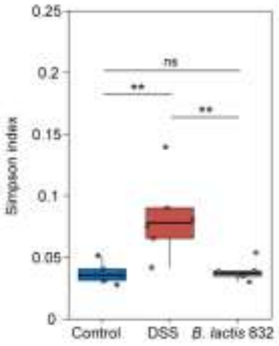

Figure S5. Alpha diversity of the gut microbiota analysis by the Simpson index (n = 6).

S6

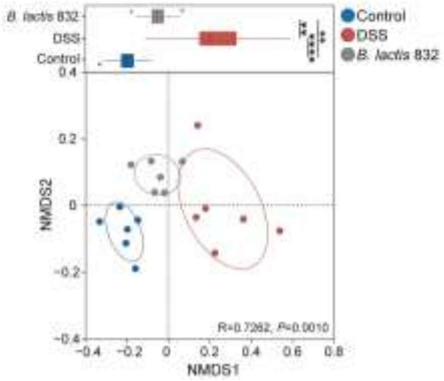

Figure S6. Non-metric Multidimensional Scaling (NMDS) and scores of the PC1 axis (upper panels) of Bray-Curtis dissimilarity based on OTUs.

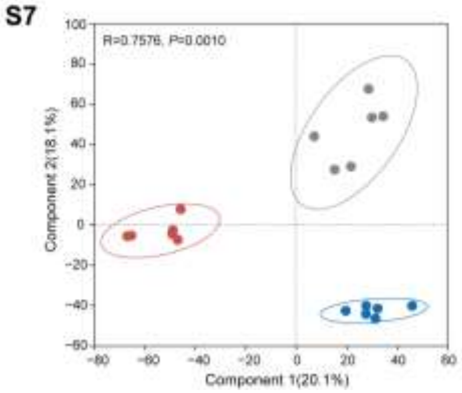

Figure S7. Partial Least Squares Discriminant Analysis (PLS-DA) illustrating changes in the composition of the fecal metabolites among Control, DSS and *B. lactis* 832 groups.
